# Cerebellar neural markers of susceptibility to social isolation and positive affective processing

**DOI:** 10.1007/s00429-019-01965-y

**Published:** 2019-11-07

**Authors:** Nichol M. L. Wong, Robin Shao, Jingsong Wu, Jing Tao, Lidian Chen, Tatia M. C. Lee

**Affiliations:** 1 State Key Laboratory of Brain and Cognitive Sciences, The University of Hong Kong, Hong Kong, China; 2 Laboratory of Neuropsychology, The University of Hong Kong, Hong Kong, China; 3 Department of Forensic and Neurodevelopmental Sciences, Institute of Psychiatry, Psychology and Neuroscience, King’s College London, London, UK; 4 Institute of Clinical Neuropsychology, The University of Hong Kong, Hong Kong, China; 5 Rehabilitation Medicine College, Fujian University of Traditional Chinese Medicine, Fuzhou, China; 6 Fujian University of Traditional Chinese Medicine, Fuzhou, China

**Keywords:** Loneliness, Social network, Cerebellum, Voxel-based morphometry, FMRI, Psycho-physiological interaction

## Abstract

Chronic loneliness predicts mood disturbances and onset of major depressive disorder. However, little research has examined the neural correlates of individual difference in susceptibility to perceiving loneliness. In addition, the role of cerebellum, which is heavily implicated in social, cognitive and affective processes, in loneliness is unclear. We studied 99 healthy individuals divided into susceptible, concordant and robust groups depending on whether the participant’s loneliness level was greater, comparable or less than her/his objective social isolation level. The cerebellar gray matter structure, functional activity and connectivity patterns during performing an emotion stroop task were examined. We found greater posterior and medial cerebellar volume in the susceptible group than the other groups. In addition, the posterior and medial cerebellar activities when processing positive versus neutral words exhibited significant interactive effects of both loneliness and social network, and susceptibility to isolation. Loneliness and social network also had positive effects on the right posterior cerebellar functional connectivity with the visual and premotor cortices. Our findings provide novel evidence on the intricate role of the cerebellum in loneliness and susceptibility to isolation, suggesting that socio-cognitive processes of the cerebellum in the hedonic domain may be a key mechanism underlying loneliness proneness.

**Electronic supplementary material:**

The online version of this article (10.1007/s00429-019-01965-y) contains supplementary material, which is available to authorized users.

## Introduction

Humans are social species and the social ties that humans form with each other have an evolutionary value to enhance survival (Cacioppo et al. 2014). If individuals lack the social ties and are socially isolated, they often show problems with physical and mental health, such as increased cardiovascular health risk (Caspi et al. 2006), immune dysregulation (Pressman et al. 2005) and psychological distress (Levula et al. 2017). Conversely, an extensive social network can be a protective factor for dementia and cognitive function decline (Bennett et al. 2006). It was proposed that loneliness, the subjective negative feeling of the perception of being socially isolated, is key in explaining the detrimental impact of social isolation, such as the onset and severity of major depression (Cacioppo et al. 2010, 2015).

Although smaller social network size of an individual is often associated with higher loneliness feeling, there exists substantial individual difference in perception of loneliness even with the same level of objective social connection. Thus, there could be discordance between subjective (i.e., loneliness) and objective social connectedness, since it is not uncommon that people can still feel lonely despite having a rich social life with extensive interpersonal connections, or may not feel lonely even when living a solitary life. Limited studies have purposefully studied the individual difference in the discrepancy between social isolation and loneliness (McHugh et al. 2017), and its association with people’s cognitive and neural functions. In particular, it is postulated that susceptibility to social isolation is closely related to altered attentional neural mechanisms, with loneliness being linked with negative affective attentional biases. Lonely individuals showed reduced tendency to use positive thinking to alleviate their negative emotions (Hawkley et al. 2009). Young lonely adults also reported higher levels of trait neuroticism, which were in turn associated with increased left dorsolateral prefrontal cortex gray matter volume, suggesting altered emotion regulation functions (Kong et al. 2015).

While affect regulation has been primarily studied in relation to the functions of the prefrontal-limbic circuitries, with the lateral and medial prefrontal cortex exerting top–down regulatory influence over the subcortical limbic-striatal areas (Wu et al. 2018), the role of other brain regions that have also been implicated in both cognitive processing and emotion regulation, such as the cerebellum, is less known. The classic view of cerebellar function is that this region is primarily involved in sensori-motor coordination functions (Grodd et al. 2001). However, more recent literature suggests that the cerebellum is also critical to social and affective processing and regulation (Van Overwalle et al. 2014; Hoche et al. 2016; Guell et al. 2018), and damage to the cerebellum would lead to the ‘cerebellar cognitive affective syndrome’, characterized by deficits in executive function, disinhibition and blunted affect (Schmahmann and Sherman 1998). The cerebellum consists of ten lobules, with lobules I–V forming the anterior cerebellar lobe, lobules VI–IX forming the posterior cerebellar lobe, lobule X forming the flocculonodular lobe and the vermis dividing cerebellum medially (Molliver and O’Hearn 2001). Specifically, the vermis in the medial part of cerebellum may be particularly implicated in affective processes, and damage to this region was commonly observed in patients with affective disturbances, while the (lateral) posterior cerebellum may be mainly involved in higher cognitive functions (Exner et al. 2004; Stoodley and Schmahmann 2010; Schmahmann 2014). Patients with major depressive disorder showed reduced gray matter volume in the vermis (Beyer and Krishnan 2002), and greater functional activations within the cerebellum, along with other regions, were found when participants were viewing social rather than non-social scenes (Powers et al. 2013). According to recent evidence, the cerebellum plays a prominent role in mentalization and a moderate role in mirroring, and these social cognitive functions were mostly located in the posterior lobules spanning both the vermis and lateral hemispheres (Van Overwalle et al. 2014; Guell et al. 2018). Consistent with this, limited evidence suggests that loneliness is linked with cerebellar functions. Higher loneliness levels were associated with increased resting-state functional connectivity between the cerebellum and the postcentral gyrus and insula, even when objective social isolation was controlled for (Layden et al. 2017). While evidence on cerebellar functional activations in associate with loneliness is lacking, a body of literature suggests that the level of cerebellar activities during affective processing, such as during performing the emotional stroop task, is altered in major depressive individuals (Fitzgerald et al. 2008; Mitterschiffthaler et al. 2008; Groenewold et al. 2013).

The current study aimed to explicitly assess the association between the structure and functions of the cerebellum, and individual difference in susceptibility to perceiving loneliness, measured by taking into account both self-reported loneliness levels and objective social connections. We hypothesised that (1) alterations in cerebellar volume, particularly in the vermis and in the posterior lobules, would be observed in participants reporting greater susceptibility to social isolation. To further elucidate the implications of the affect processing and regulatory functions of the cerebellum in susceptibility to social isolation, we also examined cerebellar functional MRI activations, while participants were performing an emotion-word Stroop task. This task recruits both higher level cognitive control networks involved in response selection and executive functions, and ‘emotion networks’ implicated in affect processing and regulation (Mitterschiffthaler et al. 2008). Based on the limited existing literature, we also tentatively hypothesised that (2) the vermis and the posterior lobules of the cerebellum would exhibit altered activities and increased functional connectivity with the prefrontal cortex in relation to loneliness, particularly among those showing higher levels of susceptibility to social isolation.

## Materials and methods

### Participants

An initial sample of 102 healthy participants was recruited through local secondary schools, universities and communities. Upon screening, only individuals that were right-handed as evaluated by the Edinburgh Inventory for Handedness (Oldfield 1971), had normal or corrected-to-normal vision, and with no histories of learning impairment or major psychiatric disorders were recruited. In addition, those with a nonverbal intelligence quotient of < 80 as measured by the Test of Nonverbal Intelligence 3rd edition (TONI-3) (Brown 2003), or histories of neurological diseases, were excluded to ensure no confounding effect of cognitive impairment or neurological changes. A final sample of 99 healthy individuals (48 male) aged between 14.80 and 69.55 years was included in the current study.

This study was approved by the Institutional Ethics Review Board of the Fujian University of Traditional Chinese Medicine and was carried out in accordance with their guidelines and regulations. Signed informed consent was obtained from all participants (if ≥ 18 years) or their guardians (if < 18 years).

### Psychological measures

#### Loneliness

The 20-item UCLA Loneliness Scale was used to measure subjective loneliness in our sample (Wu et al. 2010). This scale specifically measures individuals’ subjective feeling of loneliness (i.e., perceived social isolation) rather than objective social isolation (Hawkley and Cacioppo 2010). Participants were asked to indicate how often each of the items was descriptive of them. High scores indicate greater loneliness levels.

#### Social network

The objective structure of the extensiveness of individuals’ social network was captured using the two subscales of major social ties to relatives and friends from the Lubben Social Network Scale (Lubben 1988). Participants were asked to indicate the number of relatives and friends whom they meet or talk to at least once each week, and whom they are close enough to talk to about private matters. They were also asked to indicate the frequency of conversations with the most-contacted relative and friend. Scores of the two subscales were summed to reflect the participant’s extent of social network. Higher scores reflect richer objective social network and lower objective social isolation.

#### Susceptibility to social isolation

A score for susceptibility to isolation of each individual was calculated by subtracting the standardised score of the reversed composite score for social network, which reflects the extent of objective social isolation (or lack of social network), from the standardised score of loneliness (McHugh et al. 2017). By definition, loneliness and social isolation are two different constructs and hence, the discrepancy between the standardised scores of the two constructs could be used to describe the individual’s susceptibility or robustness to social isolation in perceiving loneliness. A more positive score denotes that the individual possessed higher loneliness than expected based on objective social isolation, implying that the individual is more susceptible to perceiving loneliness relative to their isolation status. In contrast, a more negative score denotes that the individual possessed lower loneliness than expected, implying that the individual is more robust to perceiving loneliness relative to their social-connectedness status. Based on the susceptibility score, the participants were divided into three groups: robust (lower third), concordant (middle third) and susceptible (upper third) participants.

### MRI data acquisition

Structural T1-weighted MRI images and task-based fMRI images were acquired on a 3T GE scanner with an 8-channel GE head coil. Structural T1-weighted MRI images of all participants were acquired using the magnetization-prepared rapid gradient-echo (MPRAGE) sequence with the following parameters: TR = 5.6 ms, TE = 1.8 ms, flip angle = 15^o^, slice thickness = 1 mm, FOV = 240 × 240 mm^2^, voxel size = 0.94 × 0.94 × 1 mm^3^, number of slices = 200, and sagittal acquisition. Task-based fMRI images of 60 participants (20 concordant, 21 robust and 19 susceptible) were acquired using T2*-weighted single-shot gradient-echo-planar imaging (EPI) sequence with the following parameters: TR = 2000 ms, TE = 30 ms, flip angle = 90°, FOV = 224 × 224 mm^2^, voxel size = 3.5 × 3.5 × 3.5 mm^3^, number of slices = 40, number of volumes = 240, and axial acquisition.

### fMRI task paradigm

**Fig. 1:**
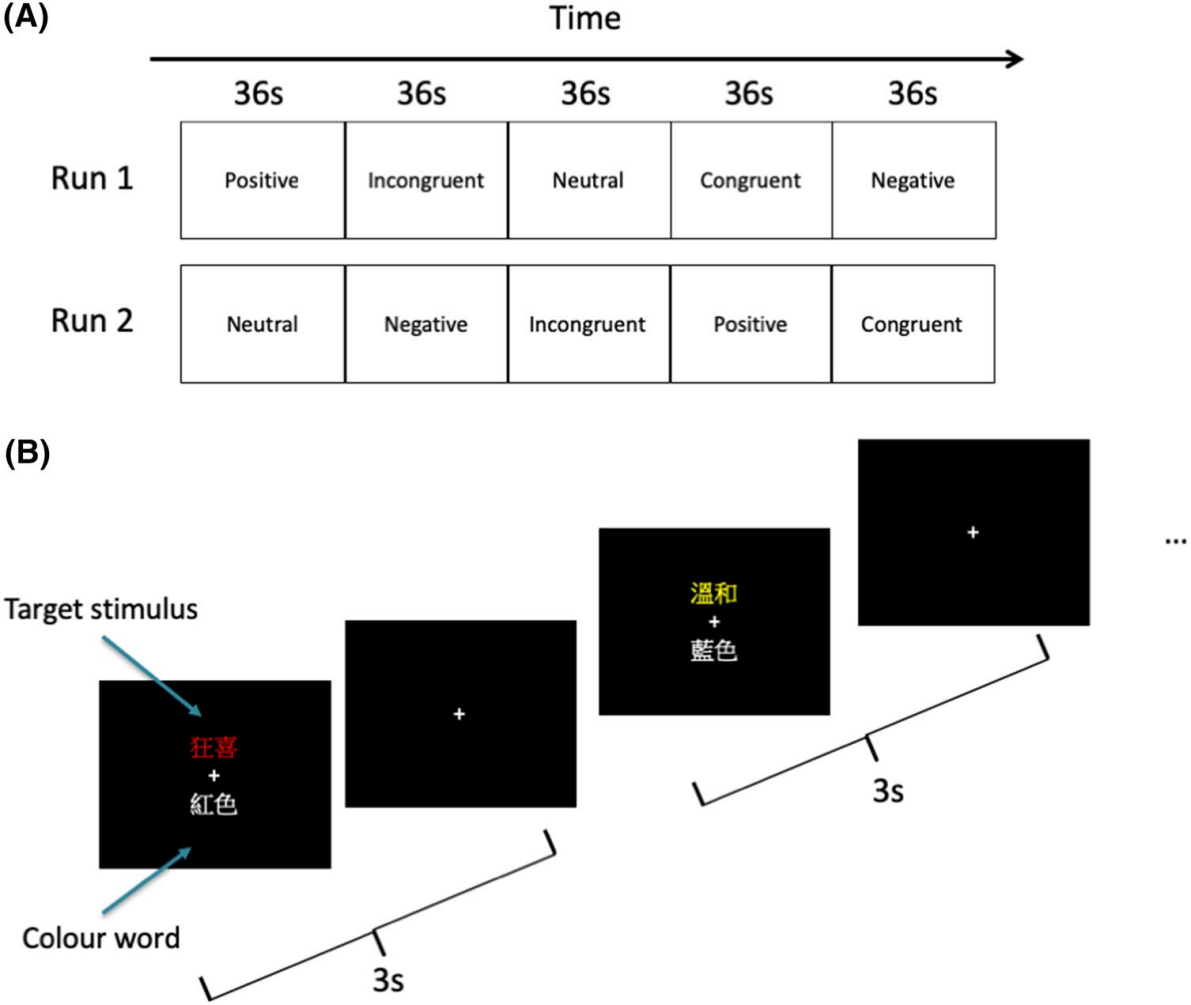
Modified emotion-word Stroop Task paradigm was administered to the participants during fMRI scanning. **a** Participants had to undergo two runs of five blocks containing different types of target stimuli with the order of blocks randomized. **b** Participants had to decide for each trial whether the meaning of the color word below the fixation cross matched the ink color of the target stimulus above the fixation cross. Each trial lasted for 3 s

The fMRI task used in this study was a two-run modified emotion-word Stroop with each run comprising five blocks presented in a randomized order (Fig. 1a). Each block consisted of 12 trials and the inter-block interval was 12 s. In each trial, a pair of word stimuli was presented above and below the fixation cross. The word above the fixation cross (i.e., target stimulus) was printed in the color of red, blue, yellow or green, and could be in any of the following conditions depending on the block (positive, negative, neutral, incongruent color or congruent color), while the word below the fixation cross was always color words printed in white (Fig. 1b). All the positive, negative and neutral words, which were translated to Chinese, were selected from the affective norms for English words database (Bradley and Lang 2007). We pre-validated the valence, arousal and frequency of usage for the translated words in a Chinese sample (see Supplementary Information for more details). In the task, participants had to decide whether the meaning of the color word presented in white below the fixation cross matched the ink color of the word presented above the fixation cross, by pressing one of two keys to indicate a ‘yes’ or ‘no’ in each trial. Trials within each block were randomized and each trial lasted for 3 s, and participants needed to respond within the first 2 s of the trial. The stimulus was displayed until the participant made the response (i.e., duration of stimuli = reaction time), after which it was replaced by the fixation cross (i.e., duration of fixation = 3 s − reaction time). The correct response was counter-balanced and participants were instructed to respond as quickly and accurately as possible. As the focus of this manuscript was on affective processing, contrasts were generated on the positive, negative, and neutral conditions only.

### MRI data analyses

The structural T1-weighted MRI images were preprocessed in MATLAB environment using the CAT12 toolbox (http://www.neuro.uni-jena.de/cat/) and SPM12 (http://www.fil.ion.ucl.ac.uk/spm/software/spm12/). Briefly, they were first segmented into three tissue classes including gray matter (GM), white matter (WM) and cerebrospinal fluid (CSF), followed by a partial volume estimation of the amount of tissue type in each voxel with the aid of the tissue probability map. high-dimensional diffeomorphic-anatomical-registration-using-exponentiated-lie-algebra (DARTEL) normalization procedure was then performed on the data (Ashburner 2007), and modulated and normalized gray matter images were generated. Subsequently, the total intracranial volume was calculated for each participant and was used as a covariate in all volumetric analyses. We focused our analyses in the cerebellum region of interest (ROI) (Figure S1) based on a priori theoretical interest and hypotheses, although whole-brain analyses were also conducted for completeness sake.

The task-based fMRI data were preprocessed using FEAT (Beckmann et al. 2006) in FSL. Each participant’s fMRI images were first corrected for motion artifact (i.e., referencing to the middle volume), spatially smoothed (full width at half maximum = 8 mm), high-pass temporal filtered (at 128 s), skull-stripped and prewhitened. The output from first-level analyses of each participant was then normalized to the Montreal Neurological Institute (MNI) standard space with 12 degrees-of-freedom, through registering to the participant’s own skull-stripped T1-weighted volume, for subsequent second-level analyses within our predefined cerebellum ROI (Figure S1).

#### Statistical analyses

Demographical and psychological differences among concordant, robust and susceptible participants were investigated using one-way analyses of variance (ANOVA). Normality of data distributions were checked by the Shapiro–Wilk test, and the bootstrapping procedure with 1000 iterations was applied to address any potential violation of the normality and/or heteroscedasticity assumption (Erceg-Hurn and Mirosevich 2008). Inter-correlations between loneliness, social network and susceptibility to social isolation within each experimental group were also explored using Spearman’s correlation.

Structurally, gray matter (GM) volumes between the three groups of participants were compared within our predefined cerebellum ROI with F-contrast using general linear model (GLM) followed by post-hoc Bonferroni-corrected pairwise comparisons. In addition, to investigate whether perceived loneliness or social network size was related to the GM volumes differently across the concordant, robust and susceptible participants, their interactive effects with the susceptibility to isolation group factor were also examined with F-contrasts using GLM. The main effects of loneliness or social network size on GM were further investigated with t-contrast using GLM when no significant interactive effects with the susceptibility to isolation group factor were revealed. For completeness, whole-brain GM analyses were also conducted.

With regard to the task-fMRI data, as our focus was on functional activities associated with affective processing, functional activations for the positive-minus-neutral, negative-minus-neutral, and positive-minus-negative contrasts were compared across the concordant, robust and susceptible groups, using F-contrasts of GLM approach within the cerebellum ROI, similar to the analyses on GM volume. To investigate whether perceived loneliness or social network size was related to the affective functional activities differently across the concordant, robust and susceptible participants, we also examined their interactive effects with the susceptibility to isolation group factor within the cerebellum ROI. Again, whole-brain functional analyses were conducted for completeness sake. To investigate the functional connectivity pattern of the cerebellar region(s), where gray matter volume or functional activations were significantly influenced by susceptibility to isolation, loneliness, social network, or their interactions, generalized psycho-physiological interaction (gPPI) analyses were also performed between the significant cerebellar area and the rest of the brain (McLaren et al. 2012). The group-level analysis models and thresholding were identical to those for the activation analyses.

All the voxelwise analyses using GLM were performed using *randomise* (Winkler et al. 2014) in FSL with 5000 permutations. The threshold-free-cluster-enhancement (TFCE) procedure was applied, adopting a familywise-error (FWE)-corrected *p*_corrected_ < 0.05 to infer significance for both the ROI and whole-brain analyses. In view of the potential confounding effects of age, gender and IQ, and the inter-correlations among loneliness, social network size and susceptibility, all subsequent analyses testing for the effect of loneliness, social network size and/or susceptibility simultaneously controlled for the other variables.

Given our participant sample encompassed a relatively wide age range, we conducted further linear regression analyses testing (1) the main effect of age on the cerebellar structure and function over all participants and within each group; (2) the interactive effect of age and group on cerebellar structure and function over all participants. These analyses additionally controlled for sex, IQ and TIV when the GM volume was the dependent measure, and controlled for sex and IQ when the functional data were the dependent measures.

## Results

### Loneliness and social network size

Based on the Shapiro–Wilk tests, only distributions of age and IQ within groups were not normal (*p* < 0.05). No group differences were detected in age, gender, education or IQ scores among the concordant, robust and susceptible groups (*p* > 0.08), confirming that the experimental groups were matched demographically. As expected, susceptible participants were significantly lonelier (*F*[2,96] = 12.443, *p* < 0.001), but at the same time had stronger social ties with family and friends (*F*[2,96] = 12.073, *p* < 0.001), compared to robust participants and concordant participants (Table 1). The concordant participants also reported greater loneliness and stronger social ties than the robust participants, but the difference did not survive post-hoc Bonferroni correction (*p* ≥ 0.058) (Table 1).

**Table 1:** Group differences in the demographics and characteristics of concordant, robust and susceptible participants

	Concordant	Robust	Susceptible	*F*	*p*
	(*n* = 33)	(*n* = 33)	(*n* = 33)		
Age (years)	32.89 (16.81)	38.72 (20.11)	28.60 (17.74)	2.551	0.083
Gender (male:female)^a^	13:20	21:12	14:19	–	0.100
Education (years)	12.13 (2.61)	13.06 (3.19)	11.55 (2.81)	2.32	0.104
TONI	103.85 (13.50)	104.70 (13.63)	103.70 (11.13)	0.058	0.943
UCLA—loneliness	37.85 (8.09)	33.36 (7.68)	43.00 (7.78)^b,c^	12.44	< 0.001
LSNS—family and friends	14.73 (4.64)	12.39 (4.35)	17.91 (4.73)^b,c^	12.07	< 0.001
Susceptibility to isolation	− 0.08 (0.23)	− 1.01 (0.44)	1.14 (0.88)	–	–

^a^Chi-squared test was performed

^b^Significantly different from concordant participants in pairwise comparison

^c^Significantly different from robust participants in pairwise comparison

The mean values are presented with standard deviation in parentheses

*LSNS* Lubben Social Network Scale, *TONI* test of nonverbal intelligence

Loneliness and social network were negatively correlated in all the groups (*ρ*[31] ≤ − 0.600, *p* < 0.001). Furthermore, loneliness (*ρ*[31] = 0.367, *p* = 0.036), but not social network (*ρ*[31] = − 0.022, *p* = 0.902), was positively related to susceptibility to social isolation in robust participants, while social network (*ρ*[31] = 0.549, *p* = 0.001), but not loneliness (*ρ*[31] = 0.219, *p* = 0.220), was positively related to susceptibility in susceptible participants.

### Susceptibility to social isolation and the structural brain

**Fig. 2:**
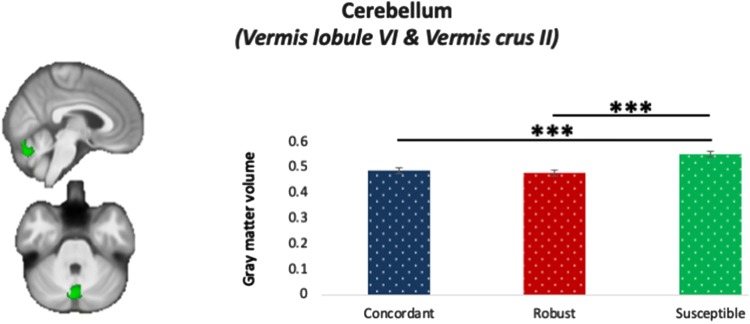
Bar chart of the significant gray matter (GM) cluster in the concordant (in blue), robust (in red), and susceptible (in green) participants with error bars ± 1 standard error. It was revealed that susceptible individuals had significantly more GM in vermis lobule VI and vermis crus II than concordant and robust individuals. ***p < 0.001

Significant group differences in GM was detected in vermis lobule VI and vermis crus II in the cerebellum (voxels = 688, maxima = [4.5, − 73.5, − 30], *p*_corrected_ = 0.012). Post-hoc Bonferroni-corrected pairwise comparisons on the average GM in the significant cerebellar cluster, adjusting for age, gender, IQ and intracranial volume, revealed that susceptible individuals showed significantly more cerebellar GM than the concordant (*M* = 0.053, SE = 0.013, *p* < 0.001) and robust individuals (*M* = 0.064, SE = 0.013, *p* < 0.001) (Fig. 2). On the other hand, no significant main effect of loneliness, or social network, was observed in the cerebellar ROI when susceptibility to isolation group factor was controlled for. In addition, no interactive effect between loneliness and susceptibility to isolation, or between social network and susceptibility to isolation, on the GM volume within the cerebellum ROI was found. No significant main or interactive effect was observed at the whole-brain level that survived TFCE correction.

### Susceptibility to loneliness and the affective functional brain

Behaviorally, no difference was observed between the concordant, robust, and susceptible participants in their accuracy or reaction time during different task conditions (Table 2).

**Table 2:** Behavioral performances of concordant, robust and susceptible participants in the emotion-word Stroop Task

	Concordant	Robust	Susceptible	*F*	*p*
	(*n* = 20)	(*n* = 21)	(*n = *19)		
Accuracy					
Positive words	0.93 (0.09)	0.93 (0.07)	0.94 (0.07)	0.02	0.98
Negative words	0.94 (0.08)	0.94 (0.09)	0.93 (0.10)	0.13	0.88
Neutral words	0.91 (0.10)	0.94 (0.04)	0.93 (0.05)	1.06	0.35
Reaction time (ms)					
Positive words	970 (160)	986 (168)	966 (175)	0.08	0.93
Negative words	962 (164)	991 (145)	1001 (175)	0.3	0.74
Neutral words	969 (160)	956 (123)	995 (212)	0.26	0.78

The mean are presented with standard deviation in parentheses

**Fig. 3:**
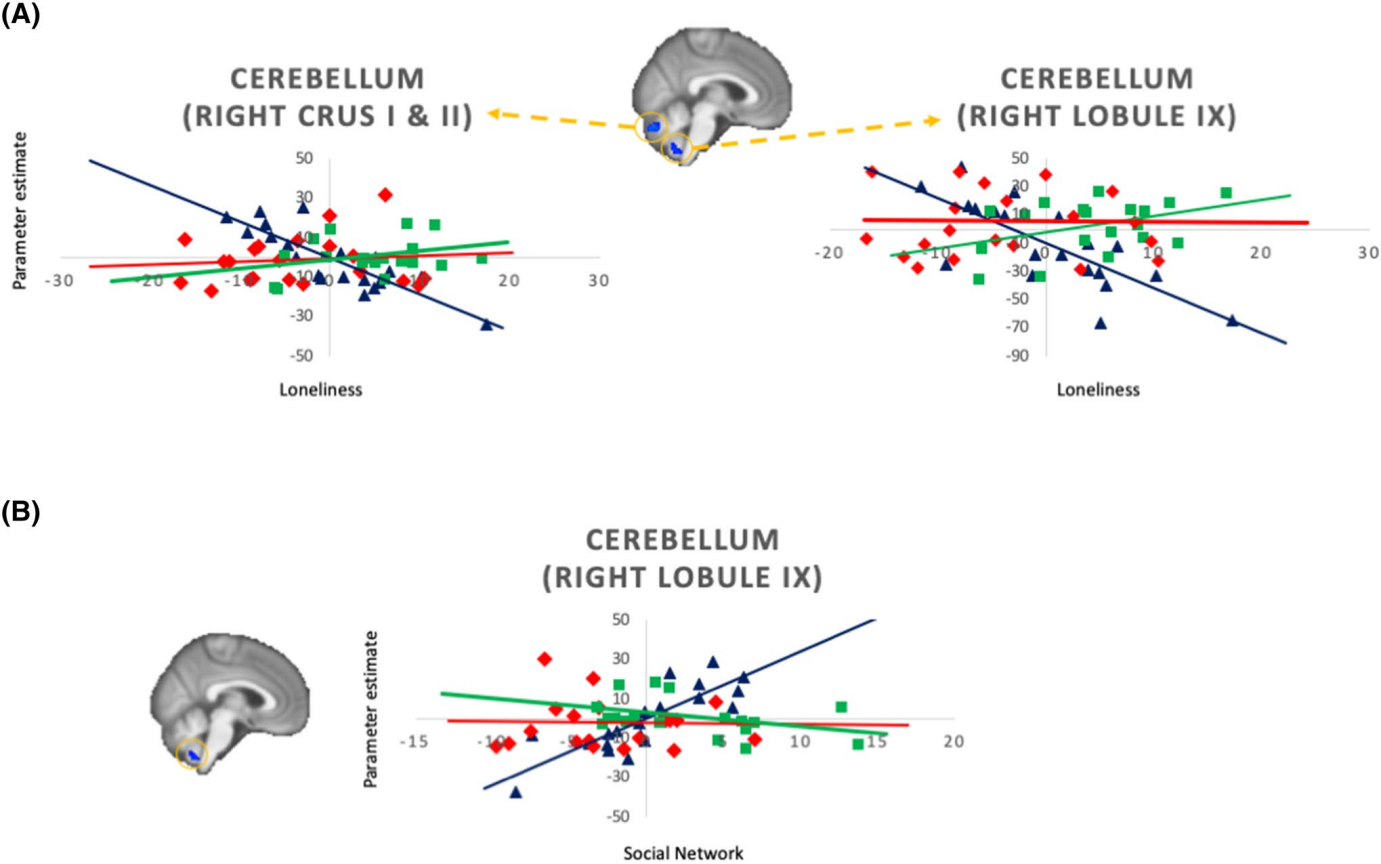
Associations between loneliness or social network size, and contrast of parameter estimates of the significant functional activations, among concordant (plotted in blue), robust (plotted in red), and susceptible (plotted in green) participants (after controlling for age, sex and IQ). It was in the *Positive*-*minus*-*Neutral* contrast we found that **a** susceptible and robust participants showed less negative associations between loneliness and cerebellar activations than concordant participants in the right crus I and II and right cerebellar lobule IX (*p* ≤ 0.001), and that **b** susceptible and robust participants showed less positive associations between social network size and cerebellar activations than concordant participants in the right cerebellar lobule IX (*p* < 0.001)

With regard to the functional activations during task-fMRI, no group differences in activations were identified in the positive-minus-neutral, negative-minus-neutral, or positive-minus-negative contrasts. However, for the positive-minus-neutral contrast, we observed interactive effects between loneliness and susceptibility to isolation on activations within the right cerebellar lobule IX, right crus I and II. We also observed interactive effects between social network and susceptibility to isolation on activations within the right cerebellar lobule IX (Table 3) (Fig. 3). Post-hoc exploratory regression analyses were then conducted to characterize these interaction results, controlling for age, sex and IQ. We observed that the associations between loneliness and functional activations in the right cerebellar lobule IX, right crus I and II were significantly less negative in the susceptible participants (*β* > 2.27, *p* < 0.001) and robust participants (*β* > 2.09, *p* ≤ 0.001) than in the concordant participants (right cerebellar lobule IX: concordant = − 1.990, robust = 0.103, susceptible = 0.286; right crus I and II: concordant = − 3.297, robust = − 0.113, susceptible = 1.041). Furthermore, the association between social network and functional activations in the right cerebellar lobule IX was significantly less positive in the susceptible participants (*β* = − 3.367, *p* < 0.001) and robust participants (*β* = − 4.086, *p* < 0.001) than in the concordant participants (right cerebellar lobule IX: concordant = 3.424, robust = 0.057, susceptible = − 0.662). On the other hand, no interactive effect between loneliness and susceptibility to isolation, or between social network and susceptibility to isolation, on the activations within the cerebellum ROI was found for negative-minus-neutral, or positive-minus-negative contrasts. Also, no significant main effect of loneliness, or social network, was observed in the cerebellar ROI when susceptibility to isolation group factor was controlled for. No significant main or interactive effect that survived TFCE correction on the functional activations was observed at the whole-brain level.

**Table 3:** Significant cerebellar clusters of functional activations showing significant interaction effects between concordant, robust and susceptible participants in the positive-minus-neutral contrast

Regions	MNI coordinates			*p*_peak_	Cluster size (voxels)
	*x*	*y*	*z*		
Association with loneliness					
Right crus I and II	16	− 76	− 30	0.023	647
Right lobule IX	4	− 54	− 56	0.039	65
Association with social network					
Right lobule IX	2	− 56	− 56	0.039	59

*p* values at peak voxel of significant clusters are reported

### Susceptibility to social isolation and the functional connectivity

**Fig. 4:**
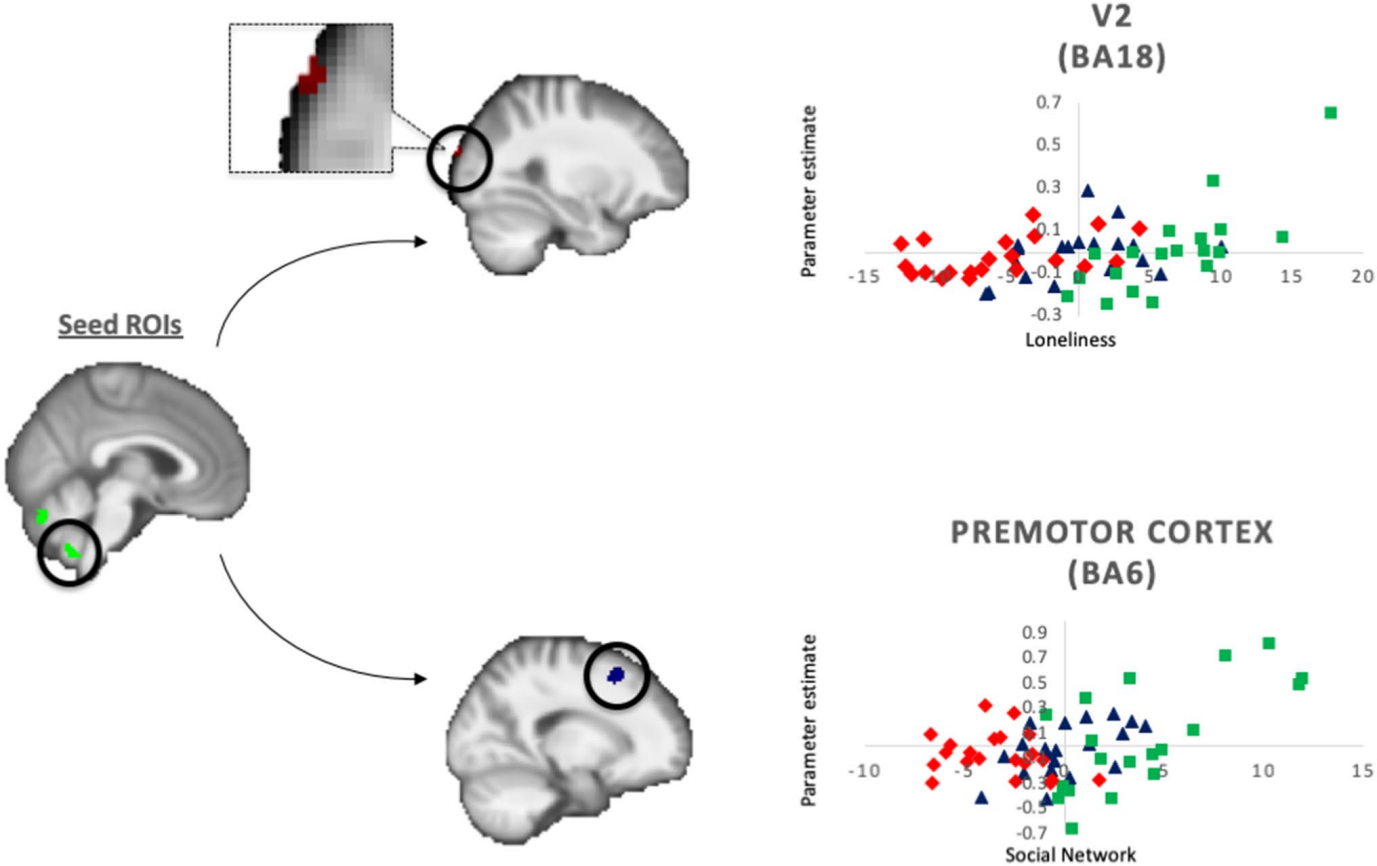
Schematic diagram of the findings from the generalized psycho-physiological interaction analyses for the positive-minus-neutral contrast across the whole brain. Seed regions of interest (ROIs) (left panel, in green) included a cluster in the vermis lobule VI, vermis crus II, right crus I and II (seed 1), and another cluster in the right cerebellar lobule IX (seed 2). Significant main effect of loneliness (*p*_corrected_ = 0.042) was identified in the functional connectivity between seed 2 and the secondary visual cortex (V2) in BA18 (top middle panel), and significant main effect of social network (*p*_corrected_ = 0.039) was identified in the functional connectivity between seed 2 and the premotor cortex in BA6. Scatterplots of the associations between perceived loneliness (upper) or social network size (lower), and contrast of parameter estimates of the significant functional connectivity adjusted for age, sex and IQ, across concordant (in blue), robust (in red), and susceptible (in green) participants were presented in the right panel

GPPI analyses for the positive-minus-neutral contrast were conducted to assess task-based functional connectivity of the seed ROIs, which were constructed based on overlapping of significant clusters from the GM volumetric and functional activation analyses. The first seed region encompassed the vermis lobule VI, vermis crus II, right crus I and II, which was derived from the overlap between the cluster with significant group differences in GM, and the cluster with significant interactive effects between loneliness and susceptibility to isolation group on task-based functional activations (Fig. 4). The second seed region included the right cerebellar lobule IX which was derived from the overlap between the cluster with significant interactive effects between loneliness and susceptibility to isolation group, and the cluster with significant interactive effects between social network and susceptibility to isolation group, on task-based functional activations (Fig. 4).

No significant main or interactive effect on the functional connectivity from the first seed ROI for the positive-minus-neutral contrast survived TFCE correction. There was also no significant interactive effect on the functional connectivity from the second seed ROI for the positive-minus-neutral contrast that survived TFCE correction. However, we observed significant positive effect of loneliness (while controlling for social network size) on the functional connectivity between the second seed ROI (i.e., right cerebellar lobule IX) and the right secondary visual cortex (BA18) (voxels = 16, maxima = [30, − 96, 22], *p*_corrected_ = 0.042), and significant positive effect of social network (while controlling for loneliness) on the functional connectivity between the second seed ROI and the right premotor cortex (BA6) (voxels = 132, maxima = [18, 18, 52], *p*_corrected_ = 0.039), for the positive-minus-neutral contrast (Fig. 4).

### Associations between age, and structural and functional brain

Finally, we examined the potential effect of age on the key cerebellar structural and functional measures. GM volume or functional measures were extracted from the significant cerebellar clusters and subjected to multiple regression analyses. We found that age was negatively associated with the GM volume in vermis lobule VI and vermis crus II over all participants (*β* = − 0.002, *p* = 0.001), and in each of the three groups (β ≤ − 0.002, *p* ≤ 0.004). However, no age × group effect was observed on any cerebellar structural or functional measures. Age also positively predicted the functional connectivity between the right cerebellar lobule IX and the right premotor cortex to the positive-minus-neutral contrast in the concordant group only (*β* = 0.007, *p* = 0.006).

## Discussion

The current study specifically examined the volumetric and functional indices of the cerebellum in relation to perceived loneliness, objective social network, and susceptibility to isolation. We found that compared to individuals reporting robust (perceiving lower level of loneliness despite relatively high social isolation) or concordant (reporting similar levels of loneliness and social isolation) pattern of susceptibility to isolation, those who were particularly vulnerable to perceiving loneliness despite relatively low social isolation exhibited increased gray matter volume of the posterior cerebellum encompassing the Crus I of the vermis and the lobule IX. Specifically during processing positive stimuli in the emotion stroop task, the association between loneliness and the activities in the right posterior cerebellum was more negative in the concordant individuals relative to both robust and susceptible individuals, while the association between social network and activities in the right posterior cerebellum was more positive among concordant individuals relative to the other two groups. Finally, during positive processing, loneliness positively predicted the right posterior cerebellum functional connectivity with the right visual cortex, and social network positively predicted the right posterior cerebellum functional connectivity with the right premotor cortex. Collectively, these findings provide novel evidence on the intricate role of the cerebellum in underlying not only the experience of loneliness, but also the susceptibility to perceiving loneliness.

A large body of clinical work indicates that damage to the posterior cerebellum is associated with cognitive deficits in executive control (e.g., planning, set-shifting, working memory), whereas vermis lesions are commonly linked to behavioural-affective disturbances (Baillieux et al. 2008; D’Angelo and Casali 2013). According to a meta-analysis, the cerebellar areas commonly activated in social cognitive tasks such as mentalization and mirroring were primarily located in the medial and posterior divisions, which also overlap extensively with the areas activated in executive control tasks (Van Overwalle et al. 2014). One possibility is that the cerebellum may not perform a domain-specific role in social cognition, but rather provides general cognitive support for complicated social processes (Van Overwalle et al. 2014). Recent theories on cerebellar functions posit that the cerebellum utilizes a ‘forward model’ based on stored associations, which uses feedback information to compute predictions and errors, which are in turn forwarded to the prefrontal and associative cortices (D’Angelo and Casali 2013). Such functions support the role of the cerebellum in prioritizing, processing and coordinating behaviours in response to incoming information, and in general cognitive and emotion control (D’Angelo and Casali 2013). Individuals with major depressive disorder, who typically exhibit deficits in both cognitive control and emotion regulation (Fitzgerald et al. 2008; Vasic et al. 2009), were found to show reduced volumes of the vermis and cerebellar atrophy (Beyer and Krishnan 2002). However, the susceptible group in the current study exhibited the opposite pattern of increased structural volumes of the posterior and medial cerebellum. Given these individuals were psychologically healthy, it could be that the increased cerebellar volume was an adaptive mechanism which compensates for the possible deficiencies in social, affective and/or cognitive functions that underlie those individuals’ high tendency to perceive loneliness despite relatively intact social network. Our current findings could not provide definitive evidence on which neural mechanisms might underlie those deficiencies, but one possibility is reduced cerebellar functional efficiency that could be related to its altered functional connectivity patterns. From a dispositional point of view, those susceptible individuals may be low on social reward dependence, a trait characterized by high sensitivity to social rewards and positive attitudes towards social relationships (Lebreton et al. 2009). Indeed, bilateral cerebellar gray matter was found to show inverse relationship with social reward dependence in the general population, which was considered to be potentially linked to the role of the cerebellum in signaling anticipation of aversive events (Ploghaus et al. 2003; Wiech et al. 2008; Lebreton et al. 2009). Thus, the increased cerebellar structural volume in loneliness-susceptible individuals may be considered as a general consequence of increased cerebellar involvement in socioaffective and cognitive processes. We also observed a negative association of age and medial cerebellar volume. This association could be primarily due to the protracted decrease of cerebellar volume which extends beyond young adulthood (Tiemeier et al. 2010; Wierenga et al. 2014). Nevertheless, the between-group difference in cerebellar volume was independent of and not moderated by the age effect.

The three participant groups performed comparably on the emotion stroop task, which might not be surprising given all participants were psychologically healthy with no neurological conditions. Thus, the observed between-group difference in brain activations during task performance was unlikely to be due to performance factors. Despite lack of main effect, we found intricate interactive effects of loneliness and susceptibility to isolation, and of social network and susceptibility to isolation. The right Crus I of lobule VII and the lobule IX were both associated with affective processing of positive and negative stimuli (Rohr et al. 2013; Keren-Happuch et al. 2014; Guell et al. 2018), whereas the Crus I and Crus II were both linked with executive functions (Van Overwalle et al. 2014; Guell et al. 2018). Our findings revealed effects of loneliness and susceptibility to isolation on posterior and medial cerebellar functions specifically in the positive word condition, highlighting the important role of hedonic processing and reward sensitivity in loneliness. Interesting, a previous study reported that patients with cerebellar stroke showed reduced pleasant experience but unchanged unpleasant experience (Turner et al. 2007), confirming the notion that the cerebellum performs essential hedonic functions. Loneliness showed particularly negative relationship with the right posterior and medial cerebellum activity to positive word stimuli in concordant participants, suggesting that for these individuals, the level of loneliness experience is primarily negatively determined by the person’s hedonic responsiveness. Notably, the cerebellum is also heavily implicated in anticipatory and attention functions (Ploghaus et al. 2003; D’Angelo and Casali 2013). Based on joint consideration of the socioaffective and cognitive processing that take place in the posterior and medial cerebellum, we speculate that individuals with increased cerebellar activity are more capable of engaging in anticipatory and consummatory hedonic processing during social contexts, which would in turn prompt those individuals to seek further social interactions, with the consequence of reduced loneliness levels. Conversely, individuals with decreased cerebellar activity might show reduced hedonic processing during social interactions, which results in decreased positive social expectation and/or processing, leading to avoidance of social interactions and elevated loneliness feeling. These explain the high contingency of loneliness feeling on social network size for the concordant participants. On the other hand, the association of loneliness and cerebellar activities to positive words was less marked and less negative in the susceptible and robust participants. It could be that the cerebellar activities in robust participants were more stable in affective responses, which might be due to greater affective regulatory and reappraisal functions in the anterior cingulate and lateral prefrontal cortices (Mohanty et al. 2007; Wiech et al. 2008; Kohn et al. 2014). As a result, the affective states and loneliness feelings of those individuals may be less reliant on the size of their social network. While we observed no significant between-group difference in prefrontal or cingulate activations during task performance, future studies that employ more socioaffectively salient tasks might shed more light on the neural functional characteristics of robust individuals. The lack of negative association between cerebellar activities to positive stimuli and loneliness in susceptible individuals might reflect low efficiency of hedonic processing in the cerebellum, and/or reduced negative affect regulatory functions (Wiech et al. 2008; D’Angelo and Casali 2013). Furthermore, the right posterior cerebellar activities to positive words were positively associated with social network size in concordant participants. Given increased social network typically entail richer social interactions and greater anticipation of social rewards, the increased cerebellar responsiveness to hedonic stimuli may reflect the individual’s generally high reward sensitivity when social support is satisfactory (Grippo et al. 2007). Such positive association was absent in susceptible and robust participants, further highlighting the potential deficits in social reward processing and responsiveness in the former group, and high stability of affect processing systems in the latter group. It should be emphasized that due to the cross-sectional nature of the current study and the lack of tasks that overtly assess social emotions, the interpretation of our findings necessarily remain speculative and should be considered with caution.

Main effects of loneliness and social network size on cerebellum functional connectivity during processing positive words in the emotion stroop task were observed, which did not differ across the susceptibility groups. Specifically, loneliness predicted more positive connectivity between the right lobule IX and the visual cortex. A meta-analysis suggested that the right lobule IX is commonly activated in emotion processing tasks, including the emotion stroop (Keren-Happuch et al. 2014). Another meta-analysis suggests that the right lobule IX is implicated in mentalizing processes (Van Overwalle et al. 2014). Moreover, recent evidence supports that the lobule IX performs both social and language functions (Guell et al. 2018). Both the posterior cerebellum and the occipital cortex were found to be activated during various executive control processes such as inhibition, conflict processing and attention (Chantiluke et al. 2012; Niendam et al. 2012), and occipital activations during performing the stroop task are commonly reported (Banich et al. 2000). A recent study reported that among individuals with autism spectrum disorder characterized by deficient social cognitive functions, resting-state functional connectivity between the cerebellum and visual areas was increased (Oldehinkel et al. 2019). Such increased connectivity may underlie increased cognitive and perceptual efforts in processing incoming social information for individuals with decreased social cognitive functions, which could be either the cause or consequence of loneliness (Hawkley and Cacioppo 2010). The premotor cortex is involved in both cognitive (e.g., attention, inhibition) and motor control functions (Abe and Hanakawa 2009). The premotor cortex also contains large populations of ‘mirror neurons’ that fire when observing other people’s actions (Heyes 2010), which are critical for inferring intention based on self-reflection. Interestingly, the posterior cerebellum is also heavily involved in mirroring processes (Van Overwalle et al. 2014), and the resting-state functional connectivity between the right cerebellum and the premotor cortex was found to be reduced in autistic children (Verly et al. 2014). Increased social network size would provide greater opportunities to observe and interact with others, which allows the individual to engage in more mirroring and theory of mind processes. Notably, both the effects of loneliness and social network size on cerebellar connectivity were independent of the susceptibility group status, indicating that the connectivity patterns were associated with actual social support and loneliness experience rather than with individual difference in proneness to perceiving loneliness. Also, both effects were observed on connectivity during processing positive words, suggesting that the brain hedonic processes, which affects the individual’s sensitivity to social rewards, may be key associates of loneliness and social support. An independent positive association between age and the cerebellar-premotor cortex connectivity was also revealed, a result that is consistent with previous literature on the development of fronto-cerebellar circuitries (Rubia et al. 2007). The implication of this age-associated change in key cerebellar connectivity remains unclear and demands future investigation.

Several limitations of the present study can be addressed in future research. We did not include tasks that explicitly assess social cognition or emotion, thus our interpretation on the role of the cerebellum in social processing remained tentative. Future studies may include mentalization, theory of mind or social emotion tasks. Second, we did not explicitly assess the participants’ state or trait affect, which could have affected the results. Future studies may additionally control for both state and trait positive and negative affects. Third, trait constructs such as empathy and social reward dependence could be measured in future studies to elucidate their relationship with susceptibility to perceiving loneliness. Finally, our participant sample encompassed a large age range, which increased the generalizability of the findings but also the heterogeneity of the participants in neural and socioaffective development. Future research should validate our findings on samples with more restricted ages.

## Conclusions

In this study, we made novel discovery on the intricate role of the posterior and medial cerebellum in underlying an individual’s proneness to perceiving loneliness, and how such proneness may further interact with loneliness and social network size in determining the cerebellar responses to positive stimuli. Our findings lay the ground for future research to more finely delineate the role of the cerebellum in loneliness, and suggests that the social cognitive and affective processing of positive stimuli may be key associate of loneliness proneness. Our findings have important implications for interventions targeted at reducing loneliness levels in the general population, which need to take into account individual differences in susceptibility to social isolation, for which the cerebellum is a putative key neural correlate.

## Electronic supplementary material

Below is the link to the electronic supplementary material.

Supplementary material 1 (DOCX 158 kb)

## References

[CR1] Abe M, Hanakawa T (2009). Functional coupling underlying motor and cognitive functions of the dorsal premotor cortex. Behav Brain Res.

[CR2] Ashburner J (2007). A fast diffeomorphic image registration algorithm. Neuroimage.

[CR3] Baillieux H, De Smet HJ, Paquier PF (2008). Cerebellar neurocognition: insights into the bottom of the brain. Clin Neurol Neurosurg.

[CR4] Banich MT, Milham MP, Atchley R (2000). fMri studies of stroop tasks reveal unique roles of anterior and posterior brain systems in attentional selection. J Cogn Neurosci.

[CR5] Beckmann CF, Jenkinson M, Woolrich MW (2006). Applying FSL to the FIAC data: model-based and model-free analysis of voice and sentence repetition priming. Hum Brain Mapp.

[CR6] Bennett DA, Schneider JA, Tang Y (2006). The effect of social networks on the relation between Alzheimer’s disease pathology and level of cognitive function in old people: a longitudinal cohort study. Lancet Neurol.

[CR7] Beyer JL, Krishnan KRR (2002). Volumetric brain imaging findings in mood disorders. Bipolar Disord.

[CR8] Bradley MM, Lang PJ, Coan JA, Allen JJB (2007). The international affective picture system (IAPS) in the study of emotion and attention. Handbook of emotion elicitation and assessement.

[CR9] Brown L, McCallum RS (2003). Test of nonverbal Intelligence: a language-free measure of cognitive ability. Handbook of nonverbal assessment.

[CR10] Cacioppo JT, Hawkley LC, Thisted RA (2010). Perceived social isolation makes me sad: 5-year cross-lagged analyses of loneliness and depressive symptomatology in the Chicago health, aging, and social relations study. Psychol Aging.

[CR11] Cacioppo JT, Cacioppo S, Boomsma DI (2014). Evolutionary mechanisms for loneliness. Cogn Emot.

[CR12] Cacioppo S, Grippo J, London S (2015). Loneliness: clinical import and interventions. Perspect Psychol Sci.

[CR13] Caspi A, Harrington H, Moffitt TE (2006). Socially isolated children 20 years later: risk of cardiovascular disease. Arch Pediatr Adolesc Med.

[CR14] Chantiluke K, Halari R, Simic M (2012). Fronto-striato-cerebellar dysregulation in adolescents with depression during motivated attention. Biol Psychiatry.

[CR15] D’Angelo E, Casali S (2013). Seeking a unified framework for cerebellar function and dysfunction: from circuit operations to cognition. Front Neural Circuits.

[CR16] Erceg-Hurn DM, Mirosevich VM (2008). Modern robust statistical methods: an easy way to maximize the accuracy and power of your research. Am Psychol.

[CR17] Exner C, Weniger G, Irle E (2004). Cerebellar lesions in the PICA but not SCA territory impair cognition. Neurology.

[CR18] Fitzgerald PB, Laird AR, Maller J, Daskalakis ZJ (2008). A meta-analytic study of changes in brain activation in depression. Hum Brain Mapp.

[CR19] Grippo AJ, Gerena D, Huang J (2007). Social isolation induces behavioral and neuroendocrine disturbances relevant to depression in female and male prairie voles. Psychoneuroendocrinology.

[CR20] Grodd W, Hülsmann E, Lotze M, Wildgruber D, Erb M (2001). Sensorimotor mapping of the human cerebellum: fMRI evidence of somatotopic organization. Hum Brain Mapp.

[CR21] Groenewold NA, Opmeer EM, de Jonge P (2013). Emotional valence modulates brain functional abnormalities in depression: evidence from a meta-analysis of fMRI studies. Neurosci Biobehav Rev.

[CR22] Guell X, Gabrieli JDE, Schmahmann JD (2018). Triple representation of language, working memory, social and emotion processing in the cerebellum: convergent evidence from task and seed-based resting-state fMRI analyses in a single large cohort. Neuroimage.

[CR23] Hawkley LC, Cacioppo JT (2010). Loneliness matters: a theoretical and empirical review of consequences and mechanisms. Ann Behav Med.

[CR24] Hawkley LC, Thisted RA, Cacioppo JT (2009). Loneliness predicts reduced physical activity: cross-sectional and longitudinal analyses. Heal Psychol.

[CR25] Heyes C (2010). Where do mirror neurons come from?. Neurosci Biobehav Rev.

[CR26] Hoche F, Guell X, Sherman JC, Vangel MG, Schmahmann JD (2016). Cerebellar contribution to social cognition. The Cerebellum.

[CR27] Keren-Happuch E, Chen S-HA, Ho M-HR, Desmond JE (2014). A Meta-analysis of cerebellar contributions to higher cognition from PET and fMRI studies. Hum Brain Mapp.

[CR28] Kohn N, Eickhoff SB, Scheller M (2014). Neural network of cognitive emotion regulation—an ALE meta-analysis and MACM analysis. Neuroimage.

[CR29] Kong X, Wei D, Li W (2015). Neuroticism and extraversion mediate the association between loneliness and the dorsolateral prefrontal cortex. Exp Brain Res.

[CR30] Layden EA, Cacioppo JT, Cappa SF (2017). Perceived social isolation is associated with altered functional connectivity in neural networks associated with tonic alertness and executive control. Neuroimage.

[CR31] Lebreton M, Barnes A, Miettunen J (2009). The brain structural disposition to social interaction. Eur J Neurosci.

[CR32] Levula A, Harré M, Wilson A (2017). Social network factors as mediators of mental health and psychological distress. Int J Soc Psychiatry.

[CR33] Lubben JE (1988). Assessing social networks among elderly populations. Fam Community Health.

[CR34] McHugh J, Kenny R, Lawlor B (2017). The discrepancy between social isolation and loneliness as a clinically meaningful metric: findings from the Irish and English longitudinal studies of ageing (TILDA and ELSA). Int J Geriatr Psychiatry.

[CR35] McLaren DG, Ries ML, Xu G, Johnson SC (2012). A generalized form of context-dependent psychophysiological interactions (gPPI): a comparison to standard approaches. Neuroimage.

[CR36] Mitterschiffthaler MT, Williams SCR, Walsh ND (2008). Neural basis of the emotional Stroop interference effect in major depression. Psychol Med.

[CR37] Mohanty A, Engels AS, Herrington JD (2007). Differential engagement of anterior cingulate cortex subdivisions for cognitive and emotional function. Psychophysiology.

[CR38] Molliver ME, O’Hearn E (2001). Organizational principles and microcircuitry of the cerebellum. Int Rev Psychiatry.

[CR39] Niendam TA, Laird AR, Ray KL (2012). Meta-analytic evidence for a superordinate cognitive control network subserving diverse executive functions. Cogn Affect Behav Neurosci.

[CR40] Oldehinkel M, Mennes M, Marquand A (2019). Altered connectivity between cerebellum, visual, and sensory-motor networks in autism spectrum disorder: results from the EU-AIMS longitudinal European autism project. Biol Psychiatry Cogn Neurosci Neuroimaging.

[CR41] Oldfield RC (1971). The assessment and analysis of handedness: the Edinburgh inventory. Neuropsychologia.

[CR42] Ploghaus A, Becerra L, Borras C, Borsook D (2003). Neural circuitry underlying pain modulation: expectation, hypnosis, placebo. Trends Cogn Sci.

[CR43] Powers KE, Wagner DD, Norris CJ, Heatherton TF (2013). Socially excluded individuals fail to recruit medial prefrontal cortex for negative social scenes. Soc Cogn Affect Neurosci.

[CR44] Pressman SD, Cohen S, Miller GE (2005). Loneliness, social network size, and immune response to influenza vaccination in college freshmen. Health Psychol.

[CR45] Rohr CS, Okon-Singer H, Craddock RC (2013). Affect and the brain’s functional organization: a resting-state connectivity approach. PLoS One.

[CR46] Rubia K, Smith AB, Taylor E, Brammer M (2007). Linear age-correlated functional development of right inferior fronto-striato-cerebellar networks during response inhibition and anterior cingulate during error-related processes. Hum Brain Mapp.

[CR47] Schmahmann JD (2014). Disorders of the cerebellum: ataxia, dysmetria of thought, and the cerebellar cognitive affective syndrome. J Neuropsychiatry Clin Neurosci.

[CR48] Schmahmann JD, Sherman JC (1998). The cerebellar cognitive affective syndrome. Brain.

[CR49] Stoodley CJ, Schmahmann JD (2010). Evidence for topographic organization in the cerebellum of motor control versus cognitive and affective processing. Cortex.

[CR50] Tiemeier H, Lenroot RK, Greenstein DK, Tran L, Pierson R, Giedd JN (2010). Cerebellum development during childhood and adolescence: a longitudinal morphometric MRI study. Neuroimage.

[CR51] Turner BM, Paradiso S, Marvel CL (2007). The cerebellum and emotional experience. Neuropsychologia.

[CR52] Van Overwalle F, Baetens K, Mariën P, Vandekerckhove M (2014). Social cognition and the cerebellum: a meta-analysis of over 350 fMRI studies. Neuroimage.

[CR53] Vasic N, Walter H, Sambataro F, Wolf RC (2009). Aberrant functional connectivity of dorsolateral prefrontal and cingulate networks in patients with major depression during working memory processing. Psychol Med.

[CR54] Verly M, Verhoeven J, Zink I (2014). Altered functional connectivity of the language network in ASD: role of classical language areas and cerebellum. NeuroImage Clin.

[CR55] Wiech K, Ploner M, Tracey I (2008). Neurocognitive aspects of pain perception. Trends Cogn Sci.

[CR56] Wierenga L, Langen M, Ambrosino S, van Dijk S, Oranje B, Durston S (2014). Typical development of basal ganglia, hippocampus, amygdala and cerebellum from age 7 to 24. Neuroimage.

[CR57] Winkler AM, Ridgway GR, Webster MA (2014). Permutation inference for the general linear model. Neuroimage.

[CR58] Wu ZQ, Sun L, Sun YH (2010). Correlation between loneliness and social relationship among empty nest elderly in Anhui rural area, China. Aging Ment Heal.

[CR59] Wu J, Geng X, Shao R (2018). Neurodevelopmental changes in the relationship between stress perception and prefrontal-amygdala functional circuitry. NeuroImage Clin.

