# Supplementary material for: Cerebellar neural markers of susceptibility to social isolation and positive affective processing
Source: Brain Struct Funct. 2019 Nov 7;224(9):3339–51. doi: 10.1007/s00429-019-01965-y (PMC6875157; doi:10.1007/s00429-019-01965-y)
Supplement: Supplementary file 1 — Supplementary material 1 (DOCX 158 kb) [file 429_2019_1965_MOESM1_ESM.docx]

**Cerebellar neural markers of susceptibility to social isolation and positive affective processing**

Nichol M.L. Wong ^1,2,3,†^, Robin Shao ^1,2,4,†^, Jingsong Wu ^5,†^, Jing Tao ^5^

Lidian Chen ^6,*^, Tatia M.C. Lee ^1,2,4,*^

^1^ State Key Laboratory of Brain and Cognitive Sciences, The University of Hong Kong, Hong Kong

^2^ Laboratory of Neuropsychology, The University of Hong Kong, Hong Kong

^3^ Department of Forensic and Neurodevelopmental Sciences, Institute of Psychiatry, Psychology & Neuroscience, King's College London, United Kingdom

^4^ Institute of Clinical Neuropsychology, The University of Hong Kong, Hong Kong

^5^ Rehabilitation Medicine College, Fujian University of Traditional Chinese Medicine, Fuzhou, China

^6^ Fujian University of Traditional Chinese Medicine, Fuzhou, China

^†^ These authors contributed equally to the work.

*** Correspondence to:**

1. Tatia M.C. Lee, Ph.D., Laboratory of Neuropsychology, The University of Hong Kong, Pokfulam Road, Hong Kong. Tel: 852-3917-8394. Email: tmclee@hku.hk
2. Lidian Chen, MD., Fujian University of Traditional Chinese Medicine, No. 1 Huatuo Road Shangjie Minhou, Fuzhou, 350122, China. Tel: 86-13305001237. Email: lidianchen87@163.com

**Supplementary information**

**Materials and Methods**

*Stimuli of fMRI task paradigm*

The fMRI task used in this study was a two-run modified emotion-word Stroop. All the positive, negative and neutral words, which were translated to Chinese, were selected from the Affective Norms for English Words database (Bradley and Lang 2007). Positive words include the Chinese translation of: romantic; bless; safe; trust; ecstasy; excited; gentle; kind; peace; comfortable; intimate. Negative words include the Chinese translation of: hell; stress; danger; failure; suffering; worried; pain; frightened; afraid; hate; grieved; sad. Neutral words include the Chinese translation of: data; equivalent; effect; division; graph; influence; subtract; year; odd number; chart; hour; month. The Chinese words that we used in the emotion stroop task were pre-validated based on established valence, arousal and use frequency measures obtained from independent sample ratings (n=57). Specifically, we have established that first, the positive words were rated substantially more positive in valence than the neutral words (t (56) = 28.68, p<.001), which were in turn rated substantially more positively than the negative words (t (56) = 25.42, p<.001). Second, both the positive and negative words were rated as substantially more arousing than the neutral words (t (56) = 10.36, p<.001 and t (56) = 13.50, p<.001). Third, we compared the usage frequency of the words according to the linguistic database by Institute of Linguistics, Academia Sinica. The usage frequency was comparable across all 3 types of words (positive vs. negative: t (11) = .64; positive vs. neutral: t (11) = -.74; negative vs. neutral: t (11) = -1.13, all p ≥ 0.28). The neutral words were additionally rated by an independent panel of clinical psychologists blind to the purpose of the study on valence, using a 7-point Likert scale ranging from 3 (very positive) to -3 (very negative). The mean affective score of the neutral words was -0.02 (SD=0.13), indicating essentially neutral affective value. Thus, we conclude that the words we included in the emotion stroop task carried the intended affective values and were considered comparable in frequency of usage.

*Region of interest selection*

We focused our gray matter volumetric and fMRI analyses in the cerebellum region of interest (ROI) based on *a-priori* theoretical interest and hypotheses. This cerebellum ROI included all the cerebellar subregions derived from FSL’s probabilistic cerebellar atlas (Diedrichsen et al. 2009) (**Figure S1**).


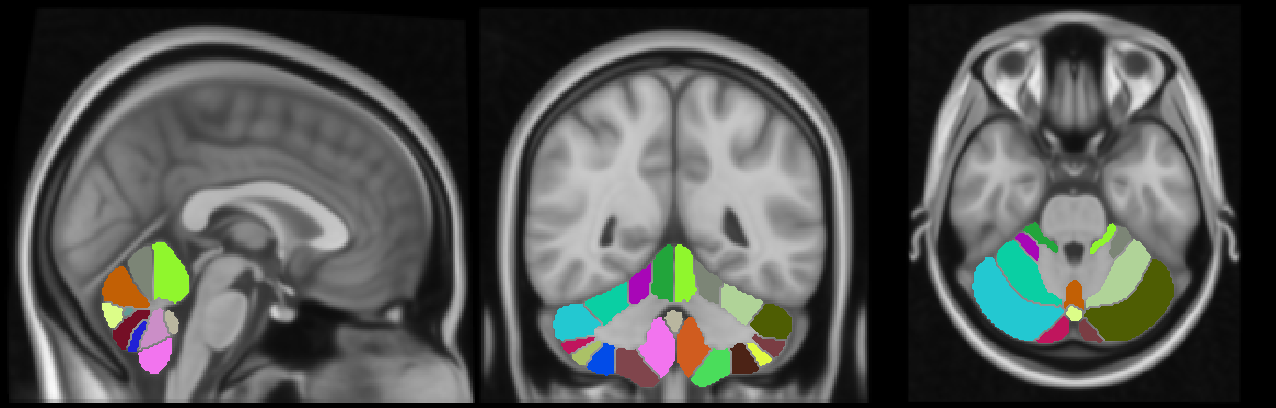


**Figure S1**. The cerebellum region of interest (ROI) mask overlain on Montreal Neurological Institute (MNI) standard template.

**References**

Bradley MM, Lang PJ (2007) The International Affective Picture System (IAPS) in the study of emotion and attention. In: Handbook of emotion elicitation and assessement. pp 29–46

Diedrichsen J, Balsters JH, Flavell J, et al (2009) A probabilistic MR atlas of the human cerebellum. Neuroimage. doi: 10.1016/j.neuroimage.2009.01.045

Huang CM, Fan YT, Lee SH, et al (in press) Cognitive reserve-mediated neural modulation of emotional interference and depressive symptoms in late-life depression. Soc Cogn Affect Neurosci
